# Prevalence of feral swine disturbance at important archaeological sites over a large landscape in Florida

**DOI:** 10.1038/srep40287

**Published:** 2017-01-10

**Authors:** Richard M. Engeman, Joseph S. Meyer, John B. Allen

**Affiliations:** 1 National Wildlife Research Center, 4101 LaPorte Ave, Fort Collins, CO 80521-2154, USA; 2 96 CEG/CEIEA, Building 696, 501 De Leon St., Eglin AFB, FL 32542, USA; 3 USDA/APHIS/WS, 2820 East University Ave., Gainesville, FL 32641, USA

## Abstract

Feral swine are globally known as one of the most destructive invasive vertebrates, damaging native habitats, native plants and animals, agriculture, infrastructure, spreading diseases. There has been little quantification on their disturbance to archaeological sites across a broad landscape. Over 6 years we inspected 293 significant archaeological sites for swine disturbance across a vast area. We found a 42% prevalence of swine disturbance among all sites, with prevalence not distinguishable among prehistoric sites, historic sites, and sites with both components. The areas of disturbance mapped within three historic homestead sites showed 5–26% of total site surface area rooted. Disturbance was not evident upon re-inspection of one of these sites after 18 months, indicating how evidence of disturbance can be obscured in this environment. Thus, our observed 42% prevalence of disturbance should be considered a minimum for disturbance occurring through time. Artifacts depths were <10 cm of the surface at 85% of the sites and <20 cm of the surface for 90% of the sites. Feral swine rooting commonly exceeds 20 cm in depth, especially in soft sandy substrates typical of Florida, making the great majority of the studied sites highly vulnerable to artifact damage or displacement.

In 1539 the Spanish explorer de Soto first introduced feral swine (*Sus scrofa*) to North America in Florida, and many additional introductions, both intentional and accidental, have followed^1^,^2^,^3^. Feral swine are now invasive to at least 35 of the 50 states in the USA^4^ and are also invasive in many countries of the world (e.g.ref. 5), and the species is considered among the 100 “World’s Worst” invaders by the IUCN Invasive Species Specialist Group^5^. This biological infamy is justified by feral swine damaging native habitats and plant species, damaging crops and livestock, preying on and competing with native animal species, posing particular threats to endangered animal and plant species^4^,^6^,^7^,^8^,^9^ as well as harboring diseases transmittable to wildlife, livestock, or humans^4^,^10^,^11^.

Like feral swine, archaeological sites are plentiful and ubiquitous throughout Florida. Archaeological sites are similarly vulnerable to swine rooting, which has been well-documented as so directly destructive to sensitive habitats and agriculture^4^,^12^. A wet climate and soft soils make much of the Florida landscape and the archaeological sites within it, vulnerable to disturbance by feral swine. Feral swine activity also accelerates erosion, further exacerbating their damage^13^. The threat for feral swine to disturb archaeological sites has been recognized in various corners of the world^12^,^14^,^15^,^16^. However there has been very little published documentation on the prevalence of feral swine disturbance to archaeological sites across an extensive landscape, nor has there been much published quantification of the scale of swine disturbance within archaeological sites.

At 187,780 ha, Eglin Air Force Base (Eglin AFB) is the largest Air Force base in the USA and approaches the size of the smallest state in the U.S., Rhode Island. Approximately 86% of Eglin AFB is forested, 12% is dedicated solely to military activities (i.e., airfields, cleared test ranges, test sites, rights-of-way, and administrative areas), and the remaining 2% is water, marshes and barrier island^17^. Feral swine are common throughout the base where they are responsible for wide-scale damage to rare and imperiled wetlands^7^. Also distributed throughout the base is a wide variety of cultural resources, with over 2800 archaeological sites identified. These sites consist of prehistoric Native American sites nearly 10,000 years-old^18^, historic homesteads and industrial sites, as well as military sites. Of the total identified cultural resources, 579 are considered significant resources based on guidelines set forth by the National Park Service and the Advisory Council on Historic Preservation. Specifically, these sites have resources listed, or evaluated as eligible, or potentially eligible for listing on the National Register of Historic Places, and all sites had been registered with the Florida State Historic Preservation Office. The Air Force is responsible for protecting these sites under various State and Federal laws and federal regulations such as the Archaeological Resource Protection Act and the National Historic Preservation Act.

The base’s stewardship obligation towards cultural sites is addressed in part by an active site monitoring program that checks site conditions throughout the year to safeguard sites when they may be impacted by a variety of land use threats, including military training activities, impacts from forestry management such as prescribed burns, and recreational use. This monitoring program was augmented in 2010 to include documentation on whether feral swine disturbance was present within each visited site, and from 2010–2016, 293 of the base’s 579 significant archaeological sites were examined for feral swine disturbance. Detailed spatial assessment of disturbance was included for three historic sites where the areas of disturbance were mapped by GPS. We report here on the results of these surveys and the implications for protecting archaeological sites in areas where feral swine occur.

## Results

The 293 significant sites surveyed represented the diversity in types of archaeological sites in the region with 104 prehistoric sites (36%), 103 historic sites (35%), and 86 sites having both prehistoric and historic components (29%). Across all sites, feral swine disturbance was observed within 122 (42%). The prevalence of swine disturbance did not vary extensively among the broad site age classifications (Χ^2^ = 3.13, df = 2, p = 0.21), with disturbance at 38% of prehistoric sites, 49% of historic sites and 38% of sites with both prehistoric and historic components (Table 1).

The archaeological records originally establishing site significance included information on artifact depth and characterizations of their landscapes and vegetation. We were particularly interested in identifying those sites most susceptible to swine disturbance based on artifact depth. Swine are known to root as deep as 1 m^18^ and easily root to 20 cm in depth^12^,^19^,^20^ and their rooting has been documented in the literature as deep as 45 cm in Florida^21^. Among the 293 sites we surveyed, 261 (90%) were known to have artifacts within 20 cm of the surface and 247 (85%) were known to have artifacts within 10 cm of the surface (Table 1). Of the 247 sites with artifacts within 10 cm of the surface, 106 (43%) were observed to have feral swine disturbance, a prevalence similar to the 38% for those sites without artifacts within 10 cm of the surface (Χ^2^ = 0.34, df = 1, p = 0.56). Of the 261 sites with artifacts within 20 cm of the surface 113 (43%) had feral swine disturbance, not distinguishable from the prevalence (30%) for sites without artifacts within 20 cm of the surface (Χ^2^ = 1.95, df = 1, p = 0.16).

**Figure 1:**
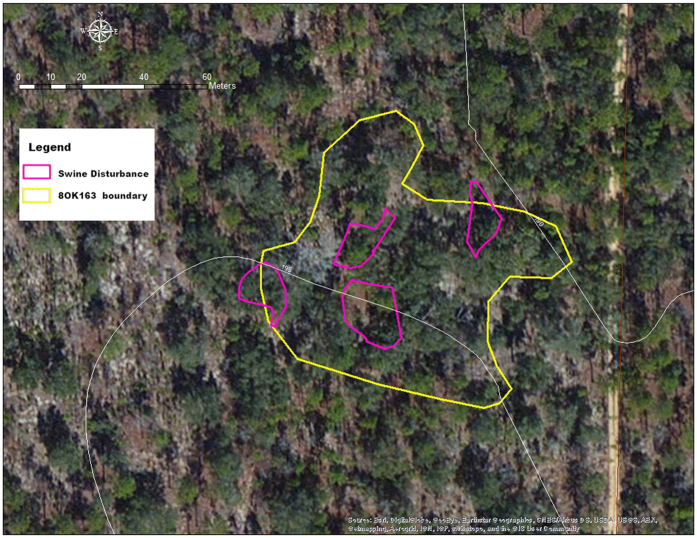
Map showing the portion of site 8OK163 disturbed by feral swine, a 1905 homestead on Eglin Air Force Base, Florida. The area of disturbance within this 5186 m^2^ site was 774 m^2^, or 15% of the total site area. The map was created using a Trimble Nomad GPS with 3 m accuracy (https://store.trimble.com/OA_HTML/ibeCCtpSctDspRte.jsp?section=10125), loaded with ArcPad 10.2 (http://www.esri.com/software/arcgis/arcpad/). Data were imported into ArcMap 10.2.2 (http://desktop.arcgis.com/en/arcmap/) to carry out calculations.

**Figure 2:**
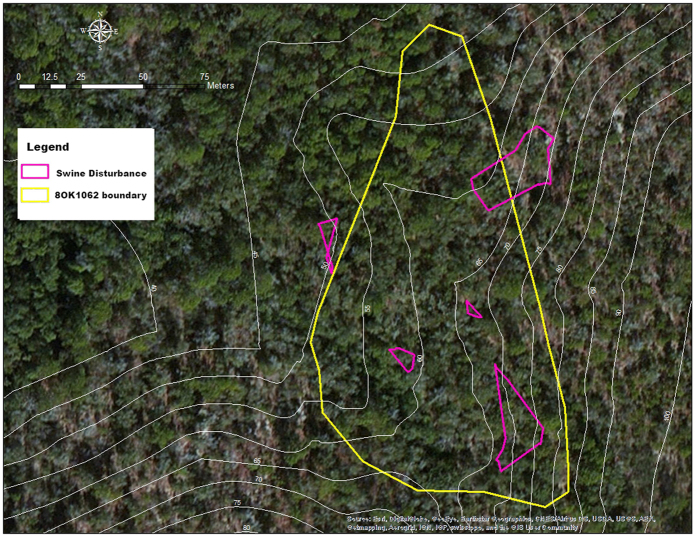
Map showing the portion of site 8OK1062 disturbed by feral swine, a 1905 homestead on Eglin Air Force Base, Florida. The area of disturbance within this 12716 m^2^ site was 648 m^2^, or 5% of the total site area. Note that one of the five mapped patches of swine disturbance was only tangential and outside the site limits and not included in calculations for area of disturbance. The map was created using a Trimble Nomad GPS with 3 m accuracy (https://store.trimble.com/OA_HTML/ibeCCtpSctDspRte.jsp?section=10125), loaded with ArcPad 10.2 (http://www.esri.com/software/arcgis/arcpad/). Data were imported into ArcMap 10.2.2 (http://desktop.arcgis.com/en/arcmap/) to carry out calculations.

**Figure 3:**
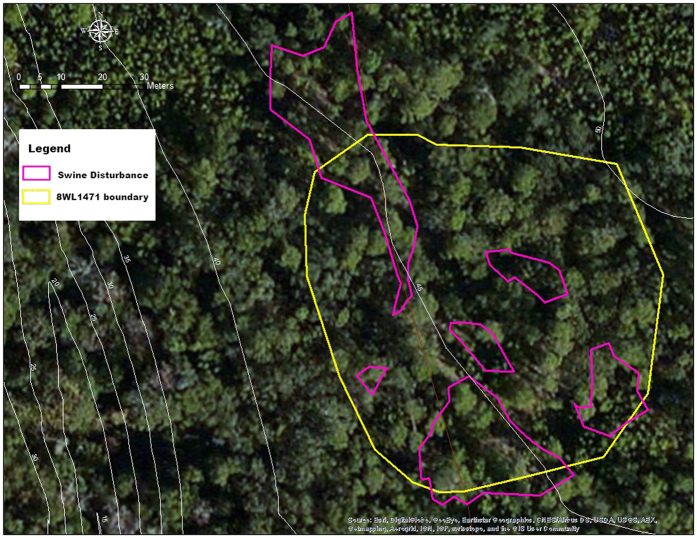
Map showing the portion of site 8WL1471 disturbed by feral swine, a 1904 homestead on Eglin Air Force Base, Florida. The area of disturbance within this 5881 m^2^ site was 1529 m^2^, or 26% of the total site area. The map was created using a Trimble Nomad GPS with 3 m accuracy (https://store.trimble.com/OA_HTML/ibeCCtpSctDspRte.jsp?section=10125), loaded with ArcPad 10.2 (http://www.esri.com/software/arcgis/arcpad/). Data were imported into ArcMap 10.2.2 (http://desktop.arcgis.com/en/arcmap/) to carry out calculations.

The 8OK163 site, a homestead claim dating to 1905, was found to have 4 patches of feral swine disturbance within its perimeter. The total site area covered 5186 m^2^, of which the area of disturbance within the site boundary totaled 774 m^2^, or 15% of the total site area (Fig. 1). Cultural material was recovered in previous archaeological site assessments as deep as 40 cm below surface (CMBS), but the majority of remains were in the upper 20 cm of deposits, making the site highly vulnerable to such disturbance. The 8OK1062 site, another 1905 homestead, had five associated areas of disturbance, although one of the disturbed patches was only tangential to and outside the site limits (Fig. 2). The combined areas of disturbance within the site limits covered 648 m^2^ (5%) of the total 12716 m^2^ of the site. Over 1100 artifacts were recovered from this site in previous archaeological site assessments, with materials recovered as deep as 40 CMBS, but most were in the upper 20 cm of deposits, also making this site highly vulnerable to disturbance. In a follow-up visit in February 2016, feral swine disturbance was not apparent due to the weathering processes on the soft, sandy soil substrate. At site 8WL1471, a homestead dating to 1904, six separate areas where the ground surface was heavily disturbed by swine were identified and measured, (Fig. 3). The combined area of the six disturbed areas within the boundary of this 5881 m^2^ site was 1529 m^2^ (26% of total site area). A total of 195 artifacts were recovered in previous archaeological site assessments between 0–60 CMBS, a majority of which were recovered from the upper 40 cm. Due to the large amount of materials on or near the surface, this site also was highly vulnerable to swine disturbance. This site was also visited March 2013 and March 2016, with feral swine disturbance observed, but not mapped on those occasions.

**Figure 4:**
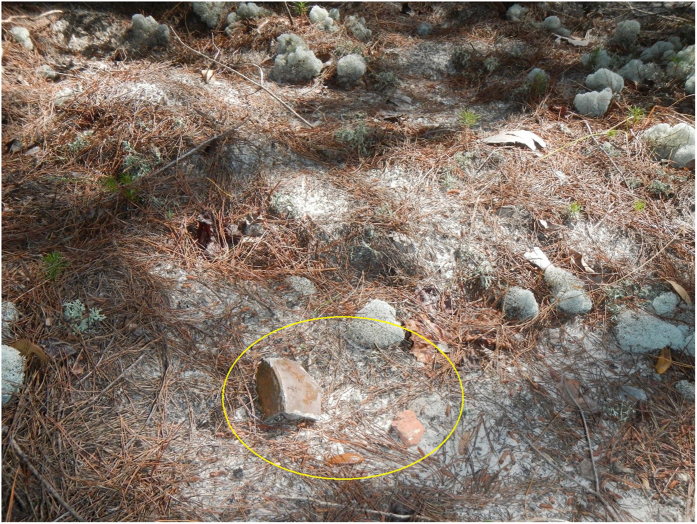
Bricks displaced (outlined) by feral swine rooting at site 8WL1471, a 1905 homestead on Eglin Air Force Base, Florida. (Photo by J. Meyer).

Each of these three sites were visited 3–7 times from 2010–2016 as part of safeguard checks on archaeological sites during base activities that might threaten them. Figure 4 shows bricks displaced by feral swine rooting at site 8WL1471 to illustrate the ease with which heavier artifacts may be moved by rooting. As an indication that feral swine disturbance in these soils can be become obscured in the wet climate, the disturbance to site 8OK1062 in September 2014 was not apparent 18 months later in February 2016. The rooting evidence indicated by disturbed soils that would show that artifacts were not in their original locations can be rapidly obscured by the weathering process in this environment. Thus, the overall 42% prevalence of feral swine disturbance we observed across all sites should be considered a minimum value.

Rooting prominently seen during the three September 2014 mapping sessions was likely due at least in part to feral swine rooting for a mast crop produced by abundant oak trees (*Quercus* spp) found at these three sites. This attractant for feral swine rooting could affect disturbance at over half of the archaeological sites on the base, because over half (52%) are characterized with oak trees within the site.

## Discussion

From June of 2010 to February 2016, we documented feral swine disturbance to archaeological sites across the breadth of Eglin AFB, and these observations provided an assessment of risk potential for feral swine to harm archaeological sites across this large area. This study consisted of two components, the first of which was to identify the presence of swine rooting and wallows within archaeological site boundaries across the base. The second component was to quantify the range of disturbance on three selected sites.

The first component identified feral swine disturbance on 42% of the significant sites on Eglin AFB. Although this is a significant percentage of the sites, this number is probably low, and the actual proportion of sites impacted by feral swine at one time or another is probably much higher, because the sandy matrix of soils on Eglin AFB appear “healed” relatively quickly through weathering in the moist climate that recurrently includes hurricanes and tropical storms. Rooting can not only be eroded to obscurity, it also can become overgrown and unrecognizable in this climate that promotes plant growth. As indicated by our observations on site 8OK1062 where old disturbance had been obscured on the ground surface after 18 months, it is very likely that sites without apparent disturbance may have had past swine disturbance erased by the weathering process. Thus, our results represented what was currently detectable and should be considered a minimal representation of accumulated damage.

The second component of our study showed that even among our three mapped sites, the amount of feral swine disturbance was highly variable ranging from 5% to 26% of the surface area of a site. If the rooting occurs within a portion of a site where artifacts are concentrated, the impact would be much greater than if rooting occurred on the edges of a site where artifact density is usually much lower.

Because swine rooting commonly exceeds 20 cm in depth (e.g.refs 12, 19, 20 and 22) and 85% of our surveyed archaeological sites had been identified as having artifacts within 10 cm of the surface, and 90% had artifacts within 20 cm of the surface, the 42% rooting prevalence across the base’s archaeological sites revealed that a substantial percentage of sites were at risk for direct swine damage to artifacts, in addition to disturbance of their artifact stratigraphy and provenience. Although feral swine are capable of rooting to a meter in depth (e.g.ref. 22), it stands to reason that rooting activity threatens archaeological sites with shallow deposits more than deeply buried sites. Nevertheless, rooting can cause erosion which could subsequently also expose the deeply buried material^13^. Rooting activity compromises the context of the deposits by displacing the remains which in turn interferes with the interpretation of the data. Furthermore, fragile material such as bone, thin glass and ceramic and flora remains could be crushed beyond recognition. Thus, rooting could cause irreparable damage to cultural deposits, especially if it occurs within shallowly deposited artifact concentrations of small sites. Eglin AFB’s archaeological sites have not been fully evaluated or excavated and it was not our intention to specifically document artifact destruction or displacement. Nevertheless, during our inspections for disturbance we could not avoid spotting where artifacts had been displaced through rooting (e.g., Fig. 4).

Archaeological sites have many facets. Site size, depth of deposits, artifact density, number and type of cultural components, types of features and other site characteristics need to be considered when evaluating site disturbance, but the key element is that feral swine clearly are impacting these nonrenewable resources and this disturbance is widespread and ongoing. Our study appears to be the first of its scale to examine the prevalence of feral swine disturbance over a large area, covering 145,000 more ha of land and over eight times as many archaeological sites as the only other study observing multiple sites for swine disturbance where 36 archaeological sites in a much smaller area in peninsular Florida^12^ were examined. Yet, despite hundreds of km separation between study areas and the discrepancy in scales for landscape area and number of sites surveyed, both studies found a 42% prevalence of feral swine disturbance among archaeological sites^12^, a striking consistency.

Feral swine cause many forms of damage to many types of resources, most of which can be mitigated, especially with the removal of feral swine. Crops can be replanted and successfully regrown the next season. Wetlands eventually can heal. Disease threats from swine can be removed with the animals. However, the provenience and stratigraphy of an archaeological site damaged by feral swine cannot regenerate with the removal of feral swine, although new damage would be averted. The passage of time is “written” in the provenience and stratigraphy of an archaeological site. Feral swine, “nature’s rototillers” (e.g.refs 23), pose a threat to destroy these timelines that can span millennia, hence “swine killing time.”

## Conclusion

This research quantified the prevalence and intensity of swine damage to National Register-eligible archeological sites over a very large area. We consider the 42% prevalence of disturbance among these important archaeological sites over such an extensive landscape to be alarming, because this prevalence likely is a minimal figure only reflecting currently observable disturbance, since we also found that significant rooting can be obscured in only 18 months. Our study covering such a large geographic scale and the smaller earlier study both showing 42% disturbance should raise awareness of the risk for feral swine to broadly disturb irreplaceable archaeological resources. Wild swine, both native and feral, are broadly distributed globally, making it likely that archaeological sites in many places in the world potentially could also suffer disturbance from their activities. These results should offer additional support for management actions towards reducing swine impacts to a landscape, in addition the well-recognized objectives of protecting natural and agricultural resources.

## Methods

### Study area

Eglin AFB is situated in the panhandle of Florida encompassing portions of Santa Rosa, Okaloosa and Walton counties covering over 187,780 ha. The main reservation is bordered by the Yellow River, Shoal River, and Titi Creek to the north, Highway 331 and private lands to the east and northeast, Choctawhatchee Bay and the Gulf of Mexico to the south, and Escambia Bay to the west. Although Eglin AFB has a variety of developed areas scattered across it, 86% of the land mass is forested with 34 natural communities.

This area of the Florida panhandle has a subtropical climate characterized by short, mild winters and warm, humid summers. The southerly latitude and its proximity to the Gulf of Mexico are controlling factors in the climate of the area. The winter temperatures typically are mild and the normal daily minimum temperature during the winter months is above freezing. On average, about 64 to 152 cm of rain falls annually. Research has documented widespread feral swine damage to unique wetland plant communities across the base^7^.

### Prevalence of disturbance among significant archaeological sites

From among the 2800 archaeological sites at Eglin AFB, we examined 293 of the 579 significant archaeological sites for evidence of feral swine disturbance (sites with resources listed, or evaluated as eligible, or potentially eligible for listing on the National Register of Historic Places). Sites were visited on the basis of need to safeguard them against planned base activities that could negatively impact them, with prime examples being military training activities, forestry management including prescribed burns, and recreational uses. Such safeguard monitoring is part of base protocol to fulfill required stewardship legal obligations as set forth by the Archaeological Resource Protection Act and the National Historic Preservation Act. Beginning in June 2010 to February 2016 we also included inspection for feral swine disturbance as part of the site monitoring program, with swine disturbance identified as ground overturned from rooting activity^19^. Researchers experienced in studying feral swine damage verified swine as the responsible species from tracks.

Previous archaeological surveys had categorized sites according to whether they were prehistoric, historic, or had both, and data on depths at which artifacts could be found were also available. We used the artifact depth data to assess vulnerability of sites to swine rooting, paying particular attention to sites with artifacts known to occur within 10 cm of the surface, and those with artifacts known to occur within 20 cm of the surface.

Comparison of prevalence of swine disturbance among site age categories (prehistoric, historic, and both) was conducted using a chi-square contingency table test. Similarly, comparison of disturbance prevalence among sites with and without artifacts within 10 cm of the surface and comparison of disturbance prevalence among sites with and without artifacts within 20 cm of the surface were also conducted using chi-square contingency table test.

### Extent of within-site disturbance

For a more thorough investigation of within-site disturbance areas, we evaluated the spatial extent of swine disturbance across the geographical area of three historic homesteads. A Global Positioning System (GPS) was used to overlay a series of transects across the site area. These predetermined transects were walked and the beginning and end points of disturbed areas were marked with flagging tape. This effort clearly defined the perimeters of disturbance across a location, which were then recorded using GPS. The recorded shapes were imported into a Geographic Information System (GIS) to calculate areas of the resulting polygons. Of the three sites selected for this more intensive documentation of the feral swine disturbance, two were within Okaloosa County and the third was in Walton County. All three sites represented homesteads that date to the early 20^th^ century.

Site 8OK163 was originally identified in the 1980’s during the Eglin Historic Preservation Plan and then tested in 1998. The site likely represents the homestead claim of Horace Jones dating to 1905. This historic site is situated in level uplands at an elevation between 58 and 59.5 m above mean sea level (AMSL). Bull Creek is the closest water source and is 350 m to the east. Vegetation is slash pine forest with mixed hardwoods, with a number of small oaks noted within the site boundary.

The 1998 investigation consisted of a surface search, the excavation of 18 50 cm × 50 cm shovel tests and a metal detector sweep. One large brick concentration was identified on the surface and appeared to be the location of a structure. The investigation recovered 86 artifacts as deep as 40 CMBS, with the majority in the upper 20 cm of deposits. These artifacts indicated a 1900 to 1920 occupation date. Mr. Jones sold the property to the Union Naval Store which operated a turpentine still just to the east of this site. Site 8OK163 was determined to be eligible for listing on the National Register of Historic Places due to the unique opportunity this site presented in comparative analysis with a larger commercial enterprise of the same time frame. From June of 2010 to February of 2016, Site 8OK163 was visited on seven occasions. In September 2014, the.feral swine disturbance was mapped to document the extent of the disturbance.

Site 8OK1062 was initially discovered in 1997 and was further tested and determined eligible for listing on the National Register of Historic Places in 2005. This site, the 1905 homestead of James F. Edge, is situated on a ridge-nose that is bound to the north by the Tenmile Creek floodplain and the west by an unnamed tributary of Tenmile Creek. The site is on level uplands at the elevation of 15 m AMSL. The landform slopes sharply towards the west into an unnamed tributary of Linton Spring Branch. Vegetation is primarily sand pine forest, with live oak, laurel oak, magnolia and persimmon scattered across the site.

The larger of two brick concentrations seem to represent the main house while the smaller brick concentration may be a smoke house. Over 1100 artifacts were recovered from this site, with much of this material recovered from the upper 20 cm of soil. Site 8OK1062 was visited for this study on four occasions from June of 2010 to February of 2016. Feral swine disturbance was mapped during September 2014.

Site 8WL1471 was initially identified in 1998 during an archaeological survey along the waters of Linton Spring Branch. The site was identified when a scatter of historic material was noted on the exposed ground surface on either side of an abandoned road. At that time researchers carried out a surface search and the excavation of 26 50 cm × 50 cm shovel tests. During this investigation three brick concentrations were noted as well as a number of small depressions that were believed to represent cultural features such as a well, trash pits or privy. A total of 195 artifacts were recovered from surface and subsurface context between 0–60 CMBS, with a majority of the remains being recovered from the upper 40 cm. The artifacts indicated the site represented a homestead dating to the early 20^th^ century which may be associated with the 1904 homestead claim of James Linton. Due to the relatively high artifact density, variation in horizontal surface distribution of materials and the association with a known land claim, site 8WL1471 was listed as potentially eligible for listing on the National Register of Historic Places and must be protected until further investigations can be conducted. Site 8WL1471 was visited on three occasions during this study and was mapped during the visit of September 2014.

## Additional Information

**How to cite this article**: Engeman, R. M. *et al*. Prevalence of feral swine disturbance at important archaeological sites over a large landscape in Florida. *Sci. Rep.* **7**, 40287; doi: 10.1038/srep40287 (2017).

**Publisher's note:** Springer Nature remains neutral with regard to jurisdictional claims in published maps and institutional affiliations.

## Figures and Tables

**Table 1:** Distribution by age category of feral swine disturbance among 293 archaeological sites eligible for the U.S. National Register of Historic Places on Eglin Air Force Base.

Age category	Number observed	Number with swine disturbance (%)	Number with artifacts ≤10 cm of surface (%)	Number with artifacts ≤20 cm of surface (%)
Prehistoric	104	39 (38%)	78 (75%)	91 (88%)
Historic	103	50 (49%)	87 (87%)	88 (86%)
Prehistoric and historic components	86	33 (38%)	82 (96%)	82 (96%)
Total	293	122 (42%)	247 (85%)	261 (90%)
